# A governance framework for medical code standardization to enhance multi-institutional data quality

**DOI:** 10.1186/s12911-026-03397-1

**Published:** 2026-02-25

**Authors:** Takanori Yamashita, Shin-Ichi Shibata, Atsushi Takada, Taeko Hotta, Rieko Izukura, Dongchon Kang, Naoki Nakashima

**Affiliations:** 1 Medical Information Center, Kyushu University Hospital, 3-1-1 Maidashi, Higashi-ku, Fukuoka, 8128582 Japan; 2 Department of Clinical Chemistry and Laboratory Medicine, International University of Health and Welfare Narita Hospital, 852 Hatakeda, Narita, 2868520 Japan; 3 School of Nursing, Faculty of Medicine, University of Miyazaki, 5200 Kihara, Kiyotake-cho, Miyazaki, 8891692 Japan; 4 Institute of Health Data Infrastructure for All, 2-9-13 Ginza, Chuo-ku, Tokyo, 1040061 Japan; 5 Department of Medical Informatics, Graduate School of Medical Sciences, Kyushu University, 3-1-1 Maidashi, Higashi-ku, Fukuoka, 8128582 Japan

**Keywords:** Real-world data, Data quality management, Code standardization, Multi-institutional research

## Abstract

**Background:**

Data quality management is crucial for performing integrated analyses of medical data across multiple institutions, and mapping facility-specific local codes to standardized codes is a critical component of this process. This study aimed to improve the medical data quality of Medical Information Database Network (MID-NET®)-cooperating institutions by developing and implementing a governance framework for medical code standardization.

**Methods:**

A governance center was established at Kyushu University Hospital, which developed a differential output tool for detecting change logs in local and standardized codes. This tool was introduced to 18 MID-NET institutions to extract differences between updates and securely transfer them to the governance center. The governance procedures involved collecting and verifying mapping tables, assigning standard codes (HOT, JLAC-10, or ICD-10), and distributing updates to cooperating institutions. The full-scale operation of the governance process began in July 2020, facilitating continuous improvement in mapping accuracy and efficiency. The most optimal standardized code was proposed by medical professionals, and feedback was provided monthly to each institution.

**Results:**

After approximately 1.5 years of governance, the correct standardized code assignment rates across all cooperating institutions were 36% for drugs, 29% for laboratory tests, and 67% for diseases. These values reflected the real-world baseline of standard code utilization in MID-NET institutions, where standardized codes had not been systematically assigned prior to governance implementation. Despite the monthly proposals provided by the governance center, the increase in registrations remained modest, particularly for laboratory tests, where the JLAC-10 codes were complex, highlighting the difficulty of achieving high coverage. However, the accumulation of differential data allowed for continuous monitoring of registration status and provided insights into problems and solutions at each institution. Mechanisms for semi-automatic registration and expansion of the governance system across multiple institutions and vendors were considered to further improve registration rates.

**Conclusion:**

Maintaining high-quality data is crucial for ensuring reliable clinical collaboration and establishing a foundation for the secondary use of real-world data. This governance model provides a practical framework for data-driven projects that integrate centralized repositories with local electronic medical records, not only within MID-NET but also for other clinical research database initiatives.

**Supplementary Information:**

The online version contains supplementary material available at 10.1186/s12911-026-03397-1.

## Background

Recently, the use of real-world data (RWD)—data accumulated in electronic medical records (EMRs) in daily medical care—has been promoted worldwide, which is expected to advance promising treatments, detect side effects, predict disease onset, facilitate drug discovery, and improve the efficiency of previously unknown medical treatments [1–3]. Real-world evidence (RWE) is scientific evidence derived from RWD analysis. In the United States, RWD and RWE are mandatory for conducting post-manufacturing safety monitoring and evaluation of pharmaceuticals. The 21st Century Cures Act, enacted in 2016, emphasized the importance of leveraging RWD to support a regulatory decision of drug efficacy, including the approval of additional indications, in addition to safety evaluations. In December 2018, the U.S. Food and Drug Administration issued the “Framework for FDA’s Real-World Evidence Program” [4], which stated that the evaluation of RWE when using RWD in regulatory decision-making depends on the methodology used to generate the evidence and the reliability of RWD [5, 6]. Therefore, RWD should be of high quality and reflect the actual situation to ensure the effectiveness of RWE. However, collecting high-quality data from multiple medical institutions is costly and time consuming. Therefore, studies have been conducted to investigate current practices and challenges in data quality assessment for public health information systems and RWD [7]. In addition to improving the reporting quality of observational studies, the Strengthening the Reporting of Observational Studies in Epidemiology (STROBE) statement is an international guideline related to RWD quality control [8, 9]. However, a systematic method that clearly defines the data quality required for the use of RWD has not yet been established [10].

Since 2016, multi-institutional data-driven projects using RWD have become active in Japan. Additionally, clinical society-led database projects have been initiated, and evidence from integrated analysis has been reported [11–13]. Following the success of the Sentinel Initiative in the United States, Medical Information Database Network (MID-NET®) was launched in Japan in 2018 by the Ministry of Health, Labour and Welfare and the Pharmaceuticals and Medical Devices Agency (PMDA). MID-NET is a medical information database that collects and analyzes medical information, such as EMRs, on a large scale to promote advanced safety measures for pharmaceuticals and other products using medical big data in Japan [14, 15]. Data validation and data quality control are important for this process, with standard code mapping being particularly important for ensuring data quality within this network. Standard codes are defined and managed by academic societies and organizations. However, some codes have a complex system, which can lead to mapping discrepancies due to differences in interpretations by the person in charge at each institution. Additionally, many institutions encounter difficulty in registering standardized codes because they prioritize the management of local codes necessary for daily hospital operations. The aim of this study was to promote data-driven clinical studies using high-quality data by improving standard code quality and governance across MID-NET cooperative institutions.

### Overview of the MID-NET system

**Fig. 1:**
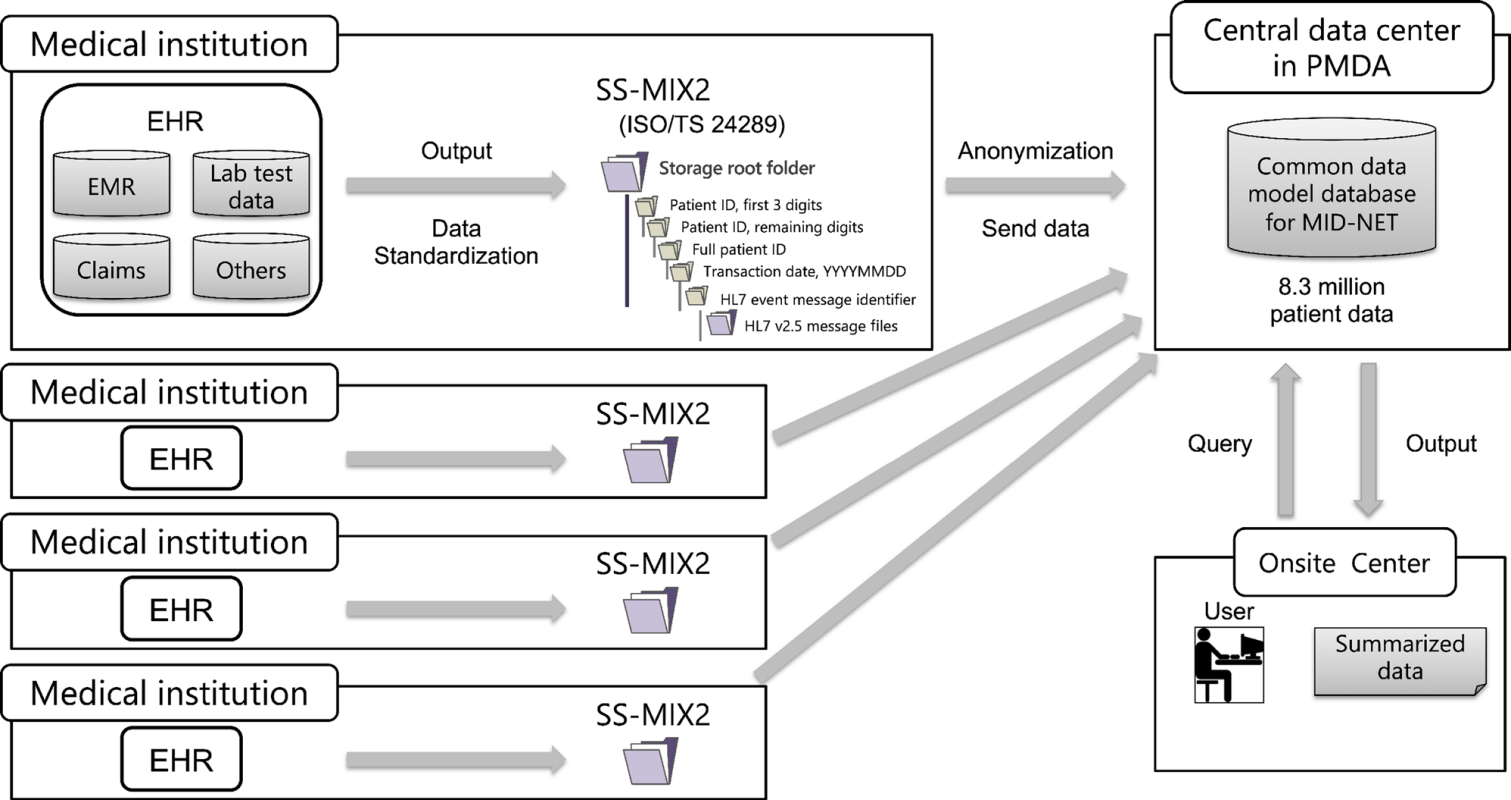
MID-NET® system overview

MID-NET employs a common data model that stores a broad spectrum of hospital information system data, including EMRs and administrative claims (Fig. 1). EMRs are a crucial component of the MID-NET system, which encompasses EMRs collected from approximately 8.3 million patients at approximately 30 medical institutions across 9 healthcare organizations. EMRs are standardized based on the message specifications of Standardized Structured Medical Information eXchange 2 (SS-MIX2) [16], which is constructed for structural standardization by extracting data from EMRs at each institution. SS-MIX2 consists of hierarchical storage of HL7 (Health Level Seven) message files, whose structure is defined in ISO/TS 24289 [17], which is constructed for structural standardization by extracting data from EMRs at each institution. The data extracted from each institution are anonymized and sent to the “common data model database” at the central data center for further analysis. Users at the on-site center, including authorized data analysts and researchers, can access and submit queries to the common data model database for remote viewing and analysis. Access authorization for the users are centrally managed by the Pharmaceuticals and Medical Devices Agency (PMDA). Subsequently, the summarized data are generated as output from the database and sent to the requestors. For each EMR of each institution participating in MID-NET, medical data with their standard codes were extracted, including patient basic information, disease names, prescription order, injection order, reception information, order information of admission, discharge, and ward change, meal, laboratory examination, bacteriological examination, radiological examination, and physiological examination, and the storage was constructed. The MID-NET database, which includes disease names, prescriptions, and laboratory values, can be used to investigate adverse drug reactions [18, 19].

MID-NET is operated under Article 15 of the Act on the Pharmaceuticals and Medical Devices Agency (Act No. 192 of 2002), which provides the legal foundation for data collection and operation. Under this legal provision, prior consent from individual patients is not required for the collection, provision, or utilization of information within MID-NET. However, MID-NET is operated with careful consideration given to the handling of sensitive information, data transfer from participating medical institutions to the central data center is conducted in accordance with legally defined procedures, data-sharing agreements, and established data-protection measures. All participating institutions follow governance requirements and internal review processes consistent with national regulations.

In this study, however, no individual patient data from MID-NET were used; only standardized medical codes (drug, laboratory test, and disease codes) were analyzed.

### Data quality management for RWD

During the construction of MID-NET, a significant deterioration in data quality was observed when linking data from EMRs to the integrated data source using SS-MIX2. The typical errors associated with this deterioration, which were confirmed at Kyushu University Hospital, included data transfer errors, standard code mapping errors, local rules for EMR at each medical site, system customization/replacement, non-numerical data (e.g., comments) included in laboratory tests, and date interpretation errors (order, execution, laboratory test, and report date). The causes of these errors are wide-ranging, including local EMR operations, EMR customization, and vendor-induced system failures. Similarly, various problems were identified at each collaborating medical institution, and the difficulty in using RWD across multiple medical institutions became evident. These medical institutions routinely introduce new drugs, laboratory reagents, and laboratory methods, and each EMR is frequently customized and updated to maximize the efficiency of medical practice. Therefore, data quality can deteriorate if data quality management activities do not continuously operate, thereby affecting the reliability of data analysis. In response to these problems, “confirmation of necessary data, confirmation of the number of data linkages, confirmation of data consistency, and confirmation of standard code mapping table” were repeatedly performed before full-scale operation to ensure high data quality. Data standardization is a key component of data quality management, and standard codes are fundamental elements of data content that facilitate this standardization.

## Methods

### Launch of a governance center and development of a code difference output tool

This study aimed to promote data-driven clinical studies using high-quality data. For this purpose, a mapping validation method was constructed, and the effectiveness of improved data quality was evaluated using a method that included the provision of centralized standard codes and validation of the mapping [20, 21]. An inter-institutional governance method was developed to enhance the quality of standard codes among MID-NET cooperative institutions [22]. From August 2020, feedback on the governance results was provided to each institution. Regarding pharmaceutical and clinical laboratory test codes, which were identified as major issues, a system and environment for confirming and proposing optimal standard codes were established, and governance was put into full-scale operation. The governance process was implemented between July 2020 and December 2021 for Japan pharmaceutical drug products reference codes (HOT) as the drug standard code [22, 23], Japan Laboratory Test Standard Code 10th Revision (JLAC-10) as the clinical laboratory standard code [22, 24, 25], and International Statistical Classification of Diseases and Related Health Problems 10th Revision (ICD-10) as the disease standard code [26, 27]. In March 2017, a governance center was established at Kyushu University Hospital, with an operational structure consisting of laboratory technicians, pharmacists, health information managers, and system engineers. The governance center specifies mapping tables and assigns standardized codes. The center developed a code difference output tool to detect daily changes in code registration at each medical institution.

The code difference output tool is an internally developed system operated within the MID-NET framework. The tool was designed to automatically compare local codes and local names with their corresponding standardized codes and standardized names in the mapping tables, and to extract daily change logs capturing differences between the previous and latest updates when any of these elements are newly registered or modified. It operates in a batch-processing manner and generates structured output files based on predefined specifications shared across all MID-NET cooperating institutions. To ensure efficient operation across heterogeneous EMR environments, the governance center defined unified specifications for the mapping tables and the output format. Based on these specifications, each EMR vendor implemented the required functionality within its MID-NET-cooperating medical institutions EMR system. Because all implementations adhered to the MID-NET specifications, functional equivalence was ensured, and each institution was able to produce consistent output formats regardless of the vendor used. As a result, the tool has been introduced to 18 MID-NET-cooperating medical institutions. Although the tool is not publicly released and therefore does not have an external URL, it was designed to be extensible and could be applied to other medical institutions outside MID-NET in future multi-institutional governance settings.

Each extracted difference is linked to the governance center through the secure MID-NET network. The tool daily checks the mapping tables and outputs the results of the comparison between local and corresponding codes at each medical site. Then, the governance center reviews the outputs and manually assigns optimal standardized codes using predefined mapping rules, master data managed by standard-setting organizations, and domain experts’ knowledge. No AI-based or automated algorithms are used in this process. The completeness of the mapping table was verified to ensure the conversion of local codes to standardized codes at each clinical site. We focused on three codes of EMR at each medical institution and determined differences in the standard codes: HOT [22, 23], JLAC-10 [22, 24, 25], and ICD-10 [26, 27]. Given that these differences, not the entire codes, were the targets of the validation process, which was labor-saving, this method enabled daily validation.

### Governance processes and procedures

**Fig. 2:**
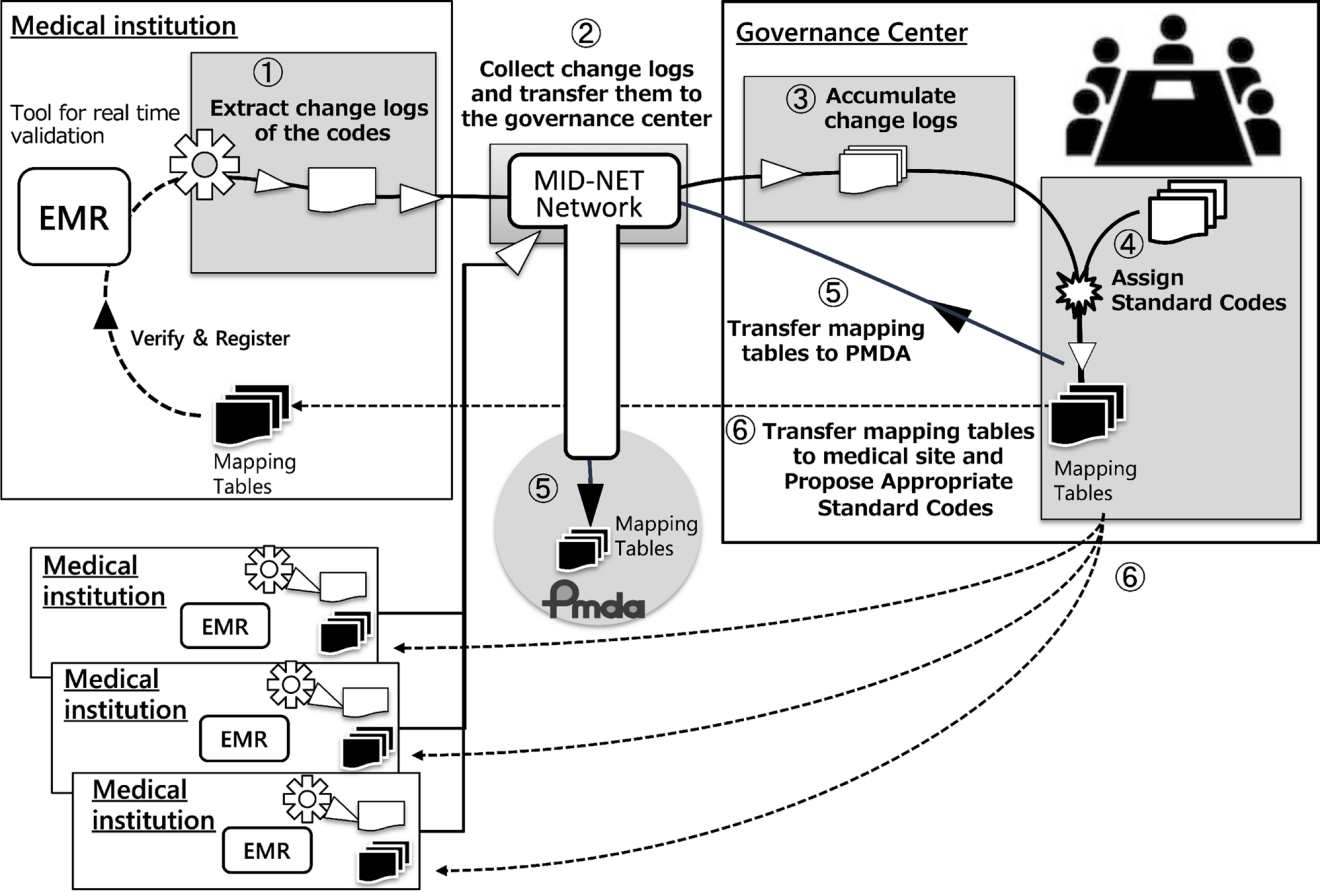
Governance center workflow for code management

The governance center used the code differential output tool and operated according to the following process (Fig. 2): extracting change logs, collecting the logs and transferring them to the governance center, accumulating the logs, assigning standard codes, transferring mapping tables to PMDA, and transferring mapping tables to every medical site. A management procedure manual was developed, based on which the governance work was implemented and recorded. The tool transferred differential data to the governance center via a dedicated network, and the differential data collected from each institution were accumulated. In this study, “standard codes” refer to the coding systems described earlier, specifically HOT for drugs, JLAC-10 for laboratory tests, and ICD-10 for diseases. The source data at each medical institution consisted of existing code values rather than free-text entries, and compared local codes with the corresponding standardized codes based on these structured code systems.

### Differential taxonomy and suggestions for standard code

**Fig. 3:**
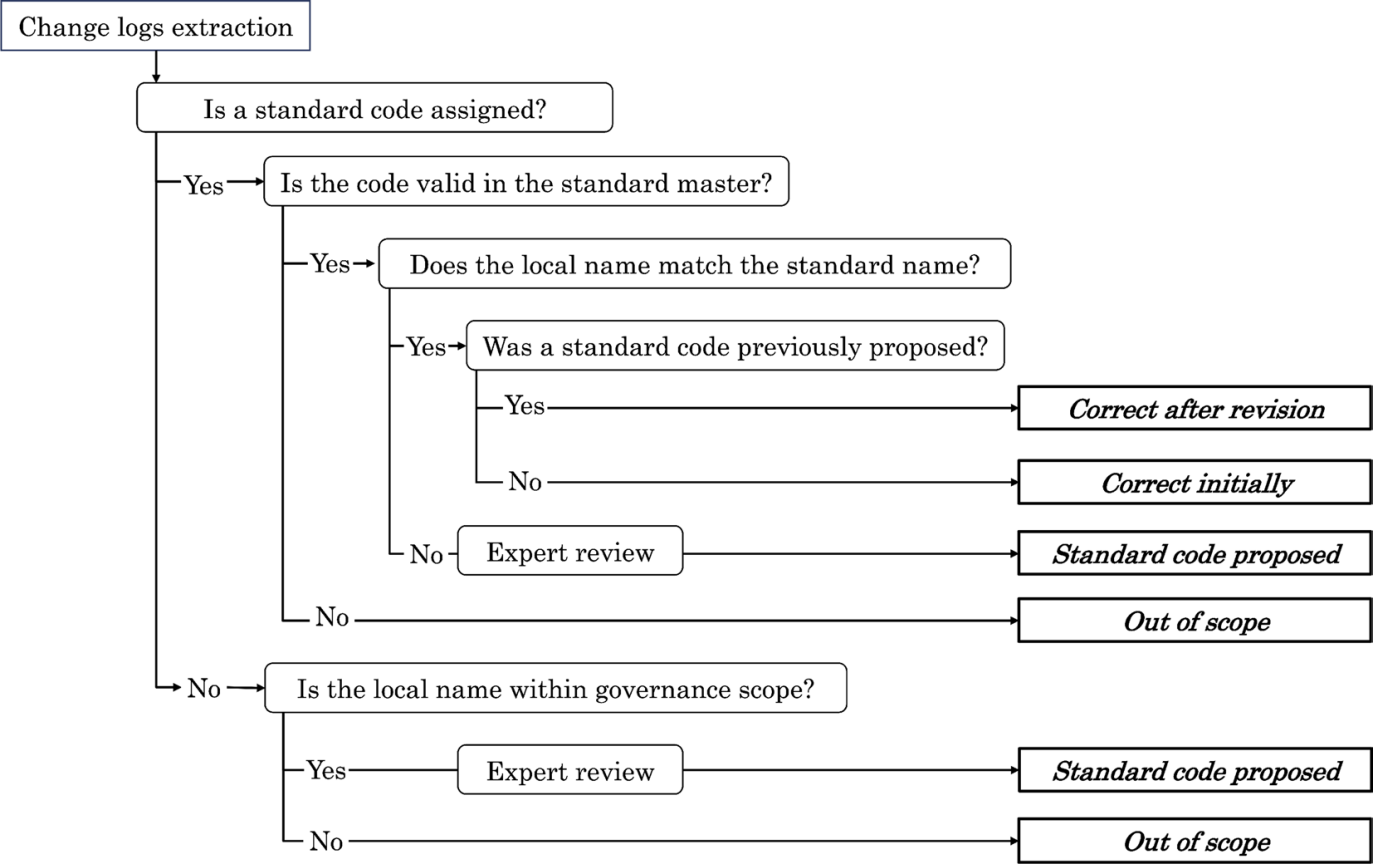
Differential taxonomy for governance of standardized code mapping

The governance taxonomy for mapping using the standard codes was defined as follows (Fig. 3):

- **Correct initially**: The local code already matched the appropriate standardized code at the time of extraction.
- **Correct after revision**: The governance center proposed a revised standardized code, and the institution subsequently implemented it.
- **Proposed standard code**: The governance center identified a mismatch and proposed the appropriate standardized code, but it had not yet been adopted by the institution at the time of assessment.
- **Out of scope**: Items that were not included within the MID-NET governance framework (e.g., codes not relevant to standardized mapping).

When a change log is extracted, the first step is to determine whether a standardized code has already been assigned. If a standardized code is present, its validity in the corresponding standard master is checked, followed by a comparison between the local name and the standardized name. If the standardized code is absent, the local item is evaluated to determine whether it falls within the scope of MID-NET governance. Items within scope are reviewed by domain experts (pharmacists, laboratory technologists, and medical information managers), and an appropriate standardized code is proposed. Items outside the governance scope are classified as out of scope. Based on these steps, each change log is classified into one of four mapping statuses: correct initially, correct after revision, proposed standard code, or out of scope.

For example, a local laboratory code labeled as “Albumin” was extracted as a change log. If a standardized code (JLAC-10) was already assigned, its presence and validity were first checked against the standard master. When the local test name and the standardized name were consistent, the mapping was classified as **Correct initially**. If a standardized code was present but the local name did not match the definition in the standard master (e.g., differences in specimen type, measurement method, or result identification), the mapping was classified as **Proposed standard code**, and expert review was conducted. Based on expert judgment, an appropriate JLAC-10 code was proposed. When the institution later updated its master data according to this proposal, the status was updated to **Correct after revision**. If no appropriate standardized code existed within the MID-NET governance framework (e.g., institution-specific test definitions), the item was classified as **Out of scope**.

## Results

The governance center collected and mapped change logs from each institution on a weekly basis using the code difference output tool. Then, the governance center proposed the optimal standard code for each institution. Once the proposed codes were registered at each institution, they were collected again as change logs, enabling the tracking of governance results over time. Therefore, governance operations were conducted and recorded in accordance with uniform management procedures. These governance activities and all code collections were conducted between July 2020 and December 2021.

**Fig. 4:**
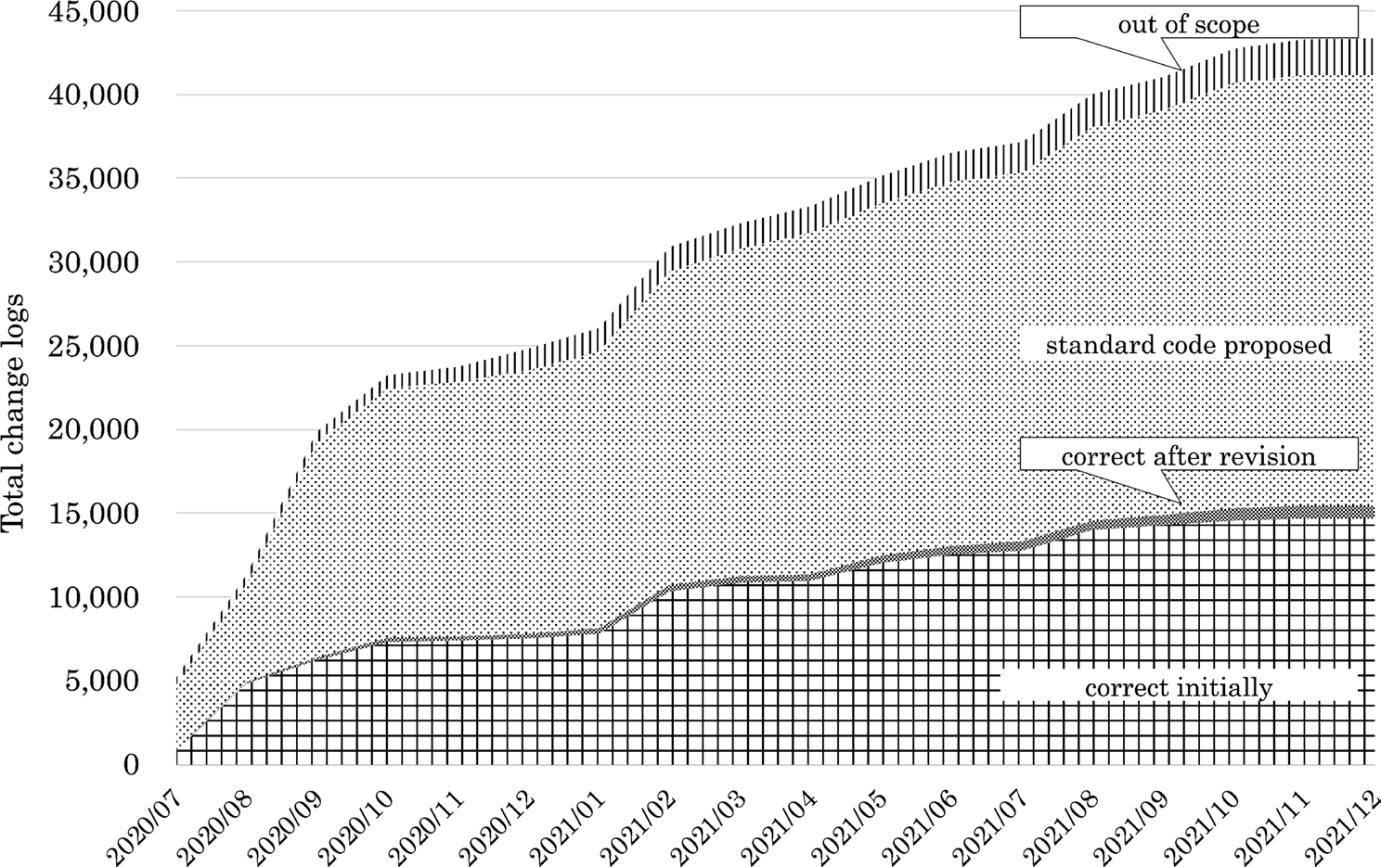
Cumulative change logs for drug codes and assigned standardized HOT codes

For drugs, 43,387 change log codes were collected, which is the largest number among the three categories. Ultimately, standardized HOT codes were assigned to 15,463 cases (Fig. 4 and Supplementary Table 1), of which each institution initially assigned codes for 14,699 cases, and an additional 764 codes were registered after the governance center proposal. Then, 25,727 codes required standardization, and 2197 codes were out of scope. Out-of-scope items included in-hospital preparations, Kampo medicines (traditional Japanese herbal medicines) [28], and medical devices.

**Fig. 5:**
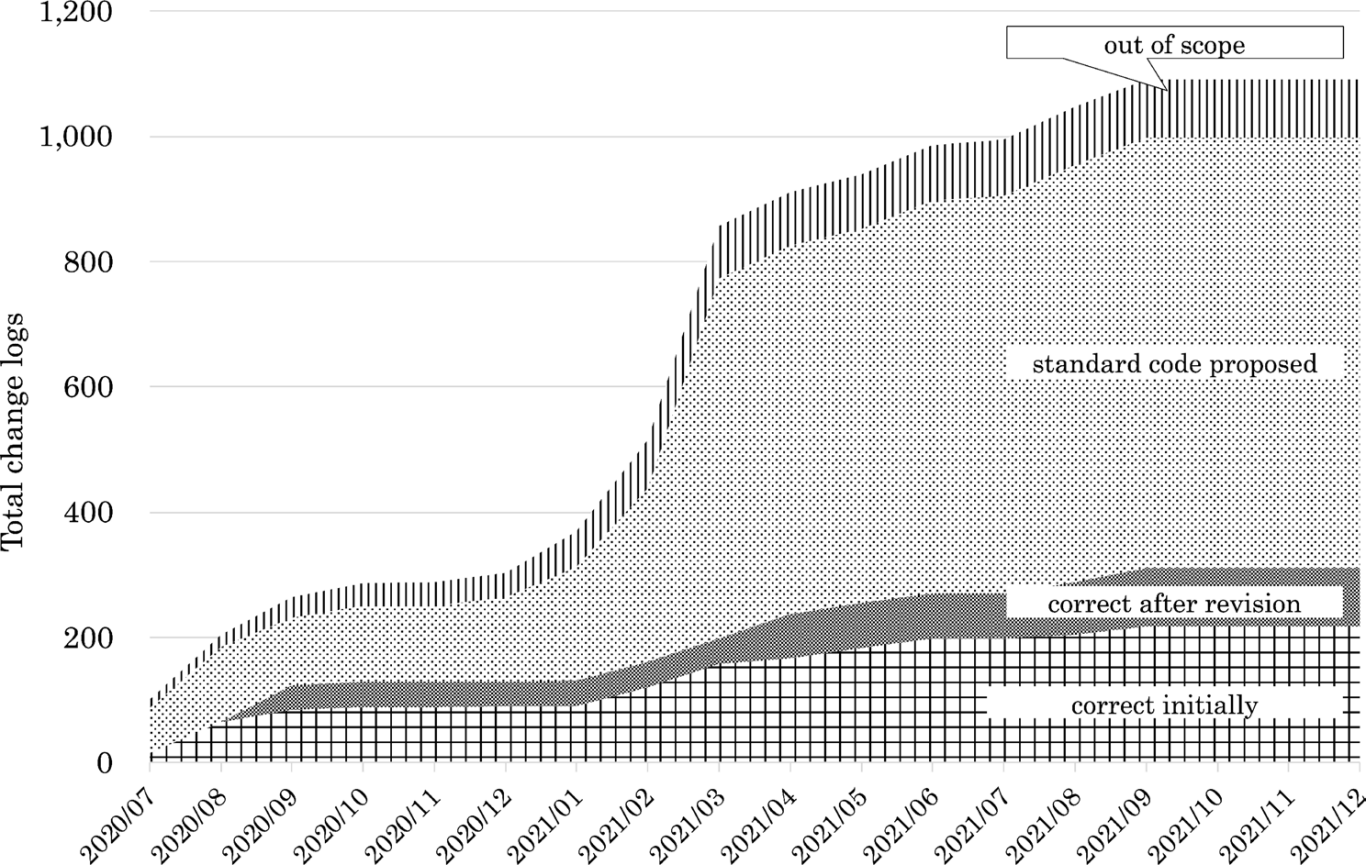
Cumulative change logs for laboratory test codes and assigned standardized JLAC-10 codes

For laboratory tests, a total of 4090 codes were collected. However, most of them were outside the scope of MID-NET. Therefore, the focus was narrowed to 1091 codes that fell within the scope of MID-NET clinical laboratory tests (Fig. 5 and Supplementary Table 2). JLAC-10 codes were ultimately assigned to 311 cases, of which each institution initially assigned 218 codes, and 93 cases were registered after the governance center proposals. Then, 687 codes required standardization, and 93 codes were out of scope. Out-of-scope items included laboratory tests that did not match the standardized master terminology.

**Fig. 6:**
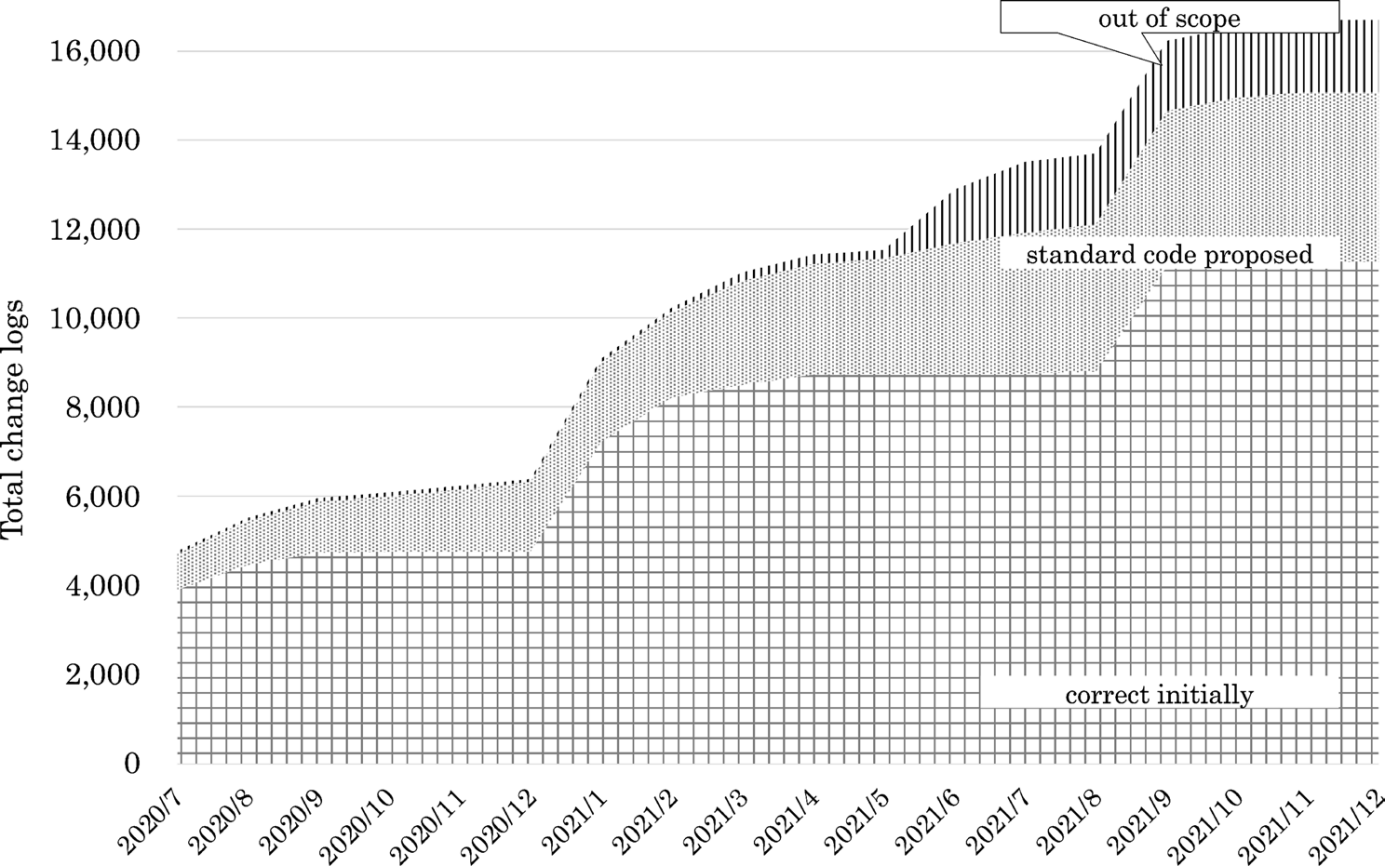
Cumulative change logs for disease codes and assigned standardized ICD-10 codes

For diseases, a total of 16,694 codes were collected. ICD-10 codes were ultimately assigned to 11,252 cases (Fig. 6 and Supplementary Table 3). Disease codes were more often correctly mapped to standardized codes from the initial stage than drug and laboratory test codes. Then, 833 codes required standardization, and 1608 codes were out of scope. Out-of-scope items mainly included modifiers and other terms not covered by the standard master.

## Discussion

### Operational challenges in the adoption of standardized codes

A system and environment for proposing optimal standardized codes were established. By accumulating differential data over time, capturing changes in master data became possible. Given that the tool was developed to provide output in a batch file, no additional work was required at the cooperating medical institutions, and the burden of generating difference files was minimal. The registration rates of standardized codes during the study period remained 36% for drugs, 29% for laboratory tests, and 67% for diseases. The governance center made monthly proposals to each institution. However, the number of standardized code registrations did not increase dramatically after these proposals. Several factors may explain why standardized code registration rates did not increase substantially despite the governance center’s proposals. One contributing factor is that standardized code assignment often requires staff with specialized coding expertise or clinical knowledge, and institutions with limited dedicated personnel may have been unable to process governance proposals promptly. In addition, institutional characteristics, such as EMR system specifications, update cycles, and local operational policies, likely affected the speed at which code revisions could be implemented. Operational priorities at many hospitals during the study period may also have shifted toward other urgent tasks, resulting in delays in updating to standardized codes. Moreover, in situations where institutions did not perceive immediate operational benefits from adopting standardized codes, the motivation to revise internal masters may have remained low. These factors collectively help explain the limited increase in registration rates observed during the study period. On the other hand, previous studies [29, 30] have reported relatively high mapping accuracy compared with the present results. However, these studies involved limited sampling and a smaller number of mapping targets. In contrast, the present study reflects real-world conditions across MID-NET cooperative institutions, including cases in which standardized codes for drugs, laboratory tests, or diseases were not pre-specified at each institution.

### Structural and governance challenges toward international interoperability

Although the ability to monitor registration rates was an achievement, the primary objective of governance was originally to increase this number, highlighting an important issue. One factor contributing to this difficulty is the structural complexity of the JLAC-10 coding system for laboratory tests. JLAC-10 codes consist of 17 digits and are defined by a combination of five elements: the analyte, identification, specimen, measurement method, and result identification. For example, a laboratory test commonly referred to as “albumin” may correspond to multiple JLAC-10 codes depending on these elements. Differences in the specimen (e.g., serum or urine), the measurement method, or the result identification can lead to distinct JLAC-10 codes, even when the local test name is identical or very similar. As a result, multiple candidate JLAC-10 codes may be proposed for a single local test name, making it difficult to uniquely identify the appropriate standardized code based solely on the test name. This multi-element structure is conceptually similar to the multi-axis representation used in LOINC, although the specific elements and code construction rules differ. Consequently, careful expert review is required to determine the most appropriate standardized code in the governance process.

From an international perspective, it is also important to position MID-NET in relation to globally adopted data models, such as the Observational Medical Outcomes Partnership (OMOP) Common Data Model. OMOP CDM is an open science community standard developed and widely used within the Observational Health Data Sciences and Informatics (OHDSI) network to support large-scale observational studies [31, 32]. MID-NET was primarily designed for use by domestic regulatory authorities in Japan and for post-marketing drug safety surveillance, with an emphasis on centralized governance, rigorous data validation, and the use of domestic standard coding systems. In contrast, OMOP was designed to support large-scale observational research and international collaboration through a standardized relational structure and globally harmonized vocabularies. Despite these differences in scope and governance, both models share a common objective: enabling consistent and reproducible analyses across institutions by standardizing heterogeneous local data. The standardized coding systems used in MID-NET can, in principle, be conceptually mapped to OMOP standard vocabularies. ICD-10 can be used directly, and drug codes defined by the HOT classification can be mapped to RxNorm. JLAC-10 laboratory test codes can be mapped to LOINC; however, this process is often complex. Differences in code granularity, measurement conditions, and code construction rules frequently necessitate one-to-many or partial mappings, requiring expert review, as discussed above.

The governance framework proposed in this study is not limited to MID-NET, but may also serve as a foundational component for OMOP-based data integration efforts in Japan. In addition, the Japanese government is progressing with its medical DX policy to build a Nationwide Health Information Platform, and has begun to consider the secondary use of healthcare data for research and international collaboration. The authors are also involved in an OMOP CDM conversion project for public databases on this platform, and the findings of the present study have been fed back into this initiative. Within this broader policy context, transitions toward internationally used common data models, such as the OMOP CDM, are being discussed as potential future directions. Although these discussions remain at a conceptual or planning stage, they highlight the growing importance of interoperability between domestic data models and international standards.

### Operational sustainability and future directions of code governance

Proposals from the governance center were conveyed to the responsible staff at each institution through direct communication, and the continuous efforts of these staff members were required to maintain the standardized code. A mechanism that semi-automatically registers the proposed standardized code to the institution master is being studied to increase the registration rate of standardized codes. Expanding the system to include multiple institutions is another challenge. Therefore, dissemination beyond system vendors and institutions requires further consideration. Maintaining the correct standardized code registration rate in real time while ensuring a certain level of quality can help provide high-quality data for each data-driven application.

The mapping tables used within the governance process did not include explicit metadata such as provenance, confidence levels, or methodological annotations. The tables primarily consisted of local codes, local name, the proposed standardized codes, standard name, and their update histories generated through the code difference output tool. Although this structure was sufficient for operational governance, incorporating transparent metadata, such as those defined in frameworks like SSSOM [33], would enhance traceability and interpretability in multi-institutional settings. Developing a metadata-enriched mapping scheme remains an important direction for future work. Assignment and operation of unique codes pose no challenges in daily medical practice at a single institution. However, when using data spanning multiple institutions, such as in MID-NET, standardized codes that can be consistently identified across all institutions are essential for ensuring the uniformity of medical data. Maintaining mapping tables to convert local codes into standardized codes is necessary in clinical settings where local codes are used, and governance of these mapping tables by experts is essential.

This study has some limitations. It was conducted at the MID-NET cooperative medical institutions. Therefore, database integration with multiple medical institutions outside the MID-NET project organizations is required to evaluate the versatility of the centralized validation system using the governance center and the code difference output tool. Particularly, factors such as the size of medical institutions, the number of staff, and EMR characteristics should be considered.

## Conclusion

The completeness of the mapping was verified to ensure that the local codes were converted to standard codes at each clinical site. The governance center assessed the completeness of the mapping tables created at each clinical site using the code difference output tool. Standard codes were applied when RWD from multiple medical sites were used. Maintaining high-quality data is crucial for ensuring robust clinical collaboration among medical sites and providing the foundation for the secondary use of RWD. The challenges of standardizing and managing codes at each medical site should be addressed. The method used for data quality management and its continuous operation offers a valuable model for data-driven projects that use a centralized data repository alongside local EMRs in any country or region. The findings of this study are valuable not only for MID-NET but also for other clinical research database projects and secondary data utilization projects that use RWD. An appropriate research design and careful consideration of the network, applications, and databases are essential for constructing a database suitable for clinical research. Only through this method can accurate data be collected and analyzed. Therefore, maintaining data quality within each organization requires innovative approaches, such as the implementation of automated mapping systems and the development of human resources.

## Electronic supplementary material

Below is the link to the electronic supplementary material.

Supplementary Material 1: Change logs for drug codes (cumulative total). It shows the cumulative total values in Fig. 3 (Cumulative change logs for drug codes and assigned standardized codes).

Supplementary Material 2: Change logs for laboratory test codes (cumulative total). It shows the cumulative total values in Fig. 4 (Cumulative change logs for laboratory test codes and assigned standardized codes).

Supplementary Material 3: Change logs for laboratory test codes (cumulative total). It shows the cumulative total values in Fig. 5 (Cumulative change logs for disease codes and assigned standardized codes).

## Data Availability

The data supporting the findings of this study are managed by the MID-NET project and were used under a contractual agreement for the purposes of this research. Therefore, these data are not publicly available. However, the data may be made available from the corresponding author upon reasonable request and with permission from the MID-NET project and the cooperative institutions.

## Abbreviations

EMR: Electronic medical records
PMDA: Pharmaceuticals and Medical Devices Agency
RWD: Real-world data
RWE: Real-world evidence
MID-NET: Medical Information Database Network
SS-MIX2: Standardized Structured Medical Information eXchange 2
HOT: Japan pharmaceutical drug products reference codes
JLAC-10: Japan Laboratory Test Standard Code 10th Revision
ICD-10: International Statistical Classification of Diseases and Related Health Problems 10th Revision
