# Supplementary material for: A governance framework for medical code standardization to enhance multi-institutional data quality
Source: BMC Med Inform Decis Mak. 2026 Feb 25;26:99. doi: 10.1186/s12911-026-03397-1 (PMC13041221; doi:10.1186/s12911-026-03397-1)
Supplement: Supplementary file 1 — Supplementary Material 1: Change logs for drug codes (cumulative total). It shows the cumulative total values in Fig. 3 (Cumulative change logs for drug codes and assigned standardized codes). [file 12911_2026_3397_MOESM1_ESM.docx]

**Additional file 1. Change logs for drug codes (cumulative total)**

| **Month/Year** | **correct initially** | **correct after revision** | **standard code proposed** | **out of scope** | **Total**  **change logs** |
| --- | --- | --- | --- | --- | --- |
| Jul 2020 | 817 | 0 | 3,923 | 498 | 5,238 |
| Aug 2020 | 4,829 | 79 | 5,806 | 579 | 11,293 |
| Sep 2020 | 6,219 | 181 | 12,840 | 632 | 19,872 |
| Oct 2020 | 7,315 | 248 | 14,901 | 765 | 23,229 |
| Nov 2020 | 7,391 | 254 | 15,210 | 889 | 23,744 |
| Dec 2020 | 7,514 | 287 | 15,718 | 1,317 | 24,836 |
| Jan 2021 | 7,830 | 322 | 16,483 | 1,422 | 26,057 |
| Feb 2021 | 10,331 | 387 | 18,727 | 1,473 | 30,918 |
| Mar 2021 | 10,822 | 399 | 19,593 | 1,531 | 32,345 |
| Apr 2021 | 10,956 | 414 | 20,356 | 1,579 | 33,305 |
| May 2021 | 12,013 | 498 | 21,007 | 1,597 | 35,115 |
| Jun 2021 | 12,534 | 541 | 21,749 | 1,726 | 36,550 |
| Jul 2021 | 12,777 | 566 | 22,014 | 1,808 | 37,165 |
| Aug 2021 | 13,989 | 595 | 23,474 | 1,953 | 40,011 |
| Sep 2021 | 14,259 | 662 | 24,138 | 1,979 | 41,038 |
| Oct 2021 | 14,584 | 706 | 25,405 | 2,026 | 42,721 |
| Nov 2021 | 14,692 | 764 | 25,700 | 2,137 | 43,293 |
| Dec 2021 | 14,699 | 764 | 25,727 | 2,197 | 43,387 |
