# Supplementary material for: A governance framework for medical code standardization to enhance multi-institutional data quality
Source: BMC Med Inform Decis Mak. 2026 Feb 25;26:99. doi: 10.1186/s12911-026-03397-1 (PMC13041221; doi:10.1186/s12911-026-03397-1)
Supplement: Supplementary file 2 — Supplementary Material 2: Change logs for laboratory test codes (cumulative total). It shows the cumulative total values in Fig. 4 (Cumulative change logs for laboratory test codes and assigned standardized codes). [file 12911_2026_3397_MOESM2_ESM.docx]

**Additional file 2. Change logs for laboratory test codes (cumulative total)**

| **Month/Year** | **correct initially** | **correct after revision** | **standard code proposed** | **out of scope** | **Total**  **change logs** |
| --- | --- | --- | --- | --- | --- |
| Jul 2020 | 16 | 0 | 72 | 13 | 101 |
| Aug 2020 | 65 | 0 | 120 | 20 | 205 |
| Sep 2020 | 84 | 39 | 109 | 33 | 265 |
| Oct 2020 | 89 | 40 | 122 | 36 | 287 |
| Nov 2020 | 89 | 40 | 122 | 38 | 289 |
| Dec 2020 | 90 | 40 | 135 | 39 | 304 |
| Jan 2021 | 91 | 40 | 182 | 57 | 370 |
| Feb 2021 | 121 | 40 | 277 | 77 | 515 |
| Mar 2021 | 158 | 40 | 575 | 84 | 857 |
| Apr 2021 | 167 | 70 | 588 | 86 | 911 |
| May 2021 | 184 | 72 | 596 | 88 | 940 |
| Jun 2021 | 198 | 72 | 628 | 88 | 986 |
| Jul 2021 | 198 | 72 | 635 | 90 | 995 |
| Aug 2021 | 205 | 83 | 667 | 92 | 1,047 |
| Sep 2021 | 218 | 93 | 687 | 93 | 1,091 |
| Oct 2021 | 218 | 93 | 687 | 93 | 1,091 |
| Nov 2021 | 218 | 93 | 687 | 93 | 1,091 |
| Dec 2021 | 218 | 93 | 687 | 93 | 1,091 |
