# Supplementary material for: A governance framework for medical code standardization to enhance multi-institutional data quality
Source: BMC Med Inform Decis Mak. 2026 Feb 25;26:99. doi: 10.1186/s12911-026-03397-1 (PMC13041221; doi:10.1186/s12911-026-03397-1)
Supplement: Supplementary file 3 — Supplementary Material 3: Change logs for laboratory test codes (cumulative total). It shows the cumulative total values in Fig. 5 (Cumulative change logs for disease codes and assigned standardized codes). [file 12911_2026_3397_MOESM3_ESM.docx]

**Additional file 3. Change logs for disease codes (cumulative total)**

| **Month/Year** | **correct initially** | **correct after revision** | **standard code proposed** | **out of scope** | **Total**  **change logs** |
| --- | --- | --- | --- | --- | --- |
| Jul 2020 | 3,896 | 0 | 811 | 69 | 4,776 |
| Aug 2020 | 4,458 | 0 | 990 | 78 | 5,526 |
| Sep 2020 | 4,740 | 0 | 1,139 | 82 | 5,961 |
| Oct 2020 | 4,742 | 0 | 1,258 | 82 | 6,082 |
| Nov 2020 | 4,748 | 0 | 1,413 | 82 | 6,243 |
| Dec 2020 | 4,749 | 0 | 1,566 | 82 | 6,397 |
| Jan 2021 | 7,223 | 0 | 1,785 | 83 | 9,091 |
| Feb 2021 | 8,223 | 0 | 1,919 | 101 | 10,243 |
| Mar 2021 | 8,482 | 1 | 2,340 | 195 | 11,018 |
| Apr 2021 | 8,734 | 1 | 2,482 | 203 | 11,420 |
| May 2021 | 8,737 | 1 | 2,594 | 207 | 11,539 |
| Jun 2021 | 8,737 | 1 | 2,950 | 1,205 | 12,893 |
| Jul 2021 | 8,745 | 1 | 3,172 | 1,580 | 13,498 |
| Aug 2021 | 8,790 | 1 | 3,321 | 1,582 | 13,694 |
| Sep 2021 | 11,138 | 1 | 3,510 | 1,587 | 16,236 |
| Oct 2021 | 11,249 | 1 | 3,694 | 1,607 | 16,551 |
| Nov 2021 | 11,252 | 1 | 3,833 | 1,607 | 16,693 |
| Dec 2021 | 11,252 | 1 | 3,833 | 1,608 | 16,694 |
